# Neuromyelitis optica spectrum disorder with increased aquaporin-4 microparticles prior to autoantibodies in cerebrospinal fluid: a case report 

**DOI:** 10.1186/s13256-018-1929-z

**Published:** 2019-01-30

**Authors:** Susanne Bejerot, Eva Hesselmark, Fariborz Mobarrez, Håkan Wallén, Max Albert Hietala, Rolf Nybom, Lennart Wetterberg

**Affiliations:** 1 Department of Clinical Neuroscience, Karolinska Institutet, SE-112 81 Stockholm, Sweden; 2 School of Medical Sciences, Örebro University, Örebro, Sweden; 3 Faculty of Medicine and Health, University Health Care Research Centre, Örebro University, Örebro, Sweden; 4 Unit of Rheumatology, Department of Medicine, Karolinska Institutet, Karolinska University Hospital, Solna, Stockholm, Sweden; 5 Karolinska Institutet, Department of Clinical Sciences, Danderyd Hospital, Division of Cardiovascular Medicine, Stockholm, Sweden

**Keywords:** Aquaporin-4, Obsessive-compulsive disorder, *La belle* indifférence, Conversion disorder, Antibodies, Microparticles, Neuromyelitis optica spectrum disorder, Pediatric autoimmune neuropsychiatric disorders, Case report

## Abstract

**Background:**

Neuromyelitis optica spectrum disorders are severe autoimmune inflammatory diseases of the central nervous system associated with the presence of immunoglobulin G antibodies against the water channel protein aquaporin-4. During exacerbation, specific aquaporin-4 immunoglobulin G may be produced intrathecally. We measured extracellular aquaporin-4 microparticles in the cerebrospinal fluid of a patient who later developed the typical symptoms and signs of a neuromyelitis optica spectrum disorder.

**Case presentation:**

A 17-year-old South American girl developed acute severe motor and vocal tics and difficulties in walking, peripheral numbness, muscle pain, and bilateral headache. At age 22, she had a multitude of motor and psychiatric symptoms. Over the years, she fulfilled the diagnostic criteria for anorexia nervosa, depression, sleep disorder, obsessive-compulsive disorder, generalized anxiety disorder, panic disorder, agoraphobia, social anxiety disorder, development coordination disorder, attention-deficit/hyperactivity disorder, hypomania, pediatric autoimmune neuropsychiatric disorders associated with streptococcal infections, conversion disorder, psychosis, and schizotypal personality syndrome. At age 24, she was found to have elevated titers of aquaporin-4 antibodies in serum, suggestive of probable neuromyelitis optica. She subsequently developed visual impairment, and swollen optic nerves were verified by magnetic resonance imaging. She was thus treated with a chimeric monoclonal antibody targeted against the pan-B-cell marker CD20 (rituximab), and almost all symptoms, including the psychiatric symptoms, rapidly decreased. We found a significant increase of extracellular microparticles of aquaporin-4 in cerebrospinal fluid sampled from our patient when she was 22 years old, 2 years before the full clinical development of neuromyelitis optica.

**Conclusions:**

Microparticles of aquaporin-4 represent subcellular arrangements that may influence the pathogenesis of neuromyelitis optica spectrum disorders and may serve as biomarkers for the underlying cellular disturbances. The increase of aquaporin-4 microparticles in cerebrospinal fluid may be used for early diagnostic purposes; for prevention; and for evaluation of effective treatment, long-term follow-up studies, and elucidating the pathophysiology in neuromyelitis optica spectrum disorders. Further studies of aquaporin-4 microparticles in cerebrospinal fluid of patients with neuromyelitis optica and similar neuropsychiatric disorders are thus called for.

**Electronic supplementary material:**

The online version of this article (https://doi.org/10.1186/s13256-018-1929-z) contains supplementary material, which is available to authorized users.

## Background

This report is about a young woman who initially presented with symptoms of a pediatric autoimmune neuropsychiatric disorder associated with streptococcal infections (PANDAS) [1] and later developed a clear diagnosis of neuromyelitis optica spectrum disorder (NMOSD). PANDAS with a chronic progressive course shares similarities with other autoimmune episodic disorders, such as multiple sclerosis (MS) and neuromyelitis optica (NMO) [2]. MS and NMO are demyelinating diseases of the central nervous system (CNS), but while MS usually has a gradual onset over time and is strongly characterized by lesions, NMO is usually characterized by an acute onset of eye pain and vision loss, as well as inflammation and lesions of the spinal cord. Also, psychiatric and cognitive impairments are frequently reported in NMO. Both MS and NMO may include loss of bowel and bladder function, limb weakness, paralysis, numbness, pain or tingling, and optic neuritis in combination with myelitis.

NMOSD are autoimmune inflammatory diseases of the CNS that mainly affect women. They are associated with serum aquaporin-4 immunoglobulin G antibodies (AQP4-IgG). Prior NMO diagnostic criteria required optic nerve and spinal cord involvement, but more restricted or more extensive CNS involvement may occur. The International Panel for NMO Diagnosis was convened to develop revised diagnostic criteria using systematic literature reviews and electronic surveys to facilitate consensus [3]. The new nomenclature defines the unifying term *neuromyelitis optica spectrum disorders* (NMOSD), which is stratified further by serologic testing (NMOSD with or without AQP4-IgG). The core clinical characteristics required for patients with NMOSD with AQP4-IgG include clinical symptoms or magnetic resonance imaging (MRI) findings related to optic nerve, spinal cord, area postrema, other brainstem regions, and diencephalic or cerebral presentations. More stringent clinical criteria, with additional neuroimaging findings, are required for diagnosis of NMOSD without AQP4-IgG or when serologic testing is unavailable.

## Case presentation

The South American patient was born at full term by cesarean section in South America and was raised by both parents. At the age of 10 years, she moved with her family to Sweden, and she attended school from arrival. When she fell ill the first time, she was performing above average in her class, had several friends, and was well integrated into Swedish society. She had no history of drug or alcohol abuse or of cigarette smoking.

There was no documented family history of encephalitis, rheumatologic or other chronic inflammatory disorders, psychiatric disorders, or tics. However, the patient’s father was diagnosed with diabetes and a maternal grandmother had a history of rheumatic fever and hypothyroidism. Also, several of the patient’s maternal cousins had been treated for recurrent tonsillitis and consequently had undergone tonsillectomy.

The patient’s early development was reported to be normal; she was a cheerful child and walked at the age of 1 year. According to the Five to Fifteen–Parent questionnaire, she showed signs of subtle motor skill problems (clumsiness, balance problems), hyperactivity, and poor concentration and possibly slight social deficits in her early school years.

- ***Age 12*** **:** *Complaints of headaches at times*
- ***Age 17:*** *Anorexia nervosa and tonsillitis*

At the age of 17 years, she started to diet to lose weight because of an alleged overweight and subsequently developed anorexia nervosa with amenorrhea. During a time course of about 6 months, she lost 15 kg with a decrease in body mass index (BMI) to 17.7 kg/m^2^. She slowly regained weight later in the year but also fell ill with fever and displayed symptoms of tonsillitis.

### Sudden onset of tics

In the afternoon of the 7th of February, at age 17, she suddenly felt numbness in the left side of her body, limb weakness, chest pain, headache, and nausea, and was admitted to the pediatric department. A pediatric neurologist did not note any signs of somatic illness explaining the symptoms. The patient’s blood pressure was normal (110/60 mmHg); she had no signs of any infection; and her C-reactive protein (CRP) was less than 1. Her electrolytes, magnesium, and liver and coagulation function (prothrombin time, activated partial thromboplastin time) were normal, and *Borrelia* serology was negative. Electroencephalography (EEG), computed tomography, and MRI (without contrast) of the brain were without any obvious pathology. A lumbar puncture did not reveal pleocytosis, nor were any oligoclonal bands identified in the cerebrospinal fluid (CSF). The following day, the patient’s headache—but not the neurological symptoms—had subsided. Two days later, when she was still in the hospital, severe gross motor tics emerged in her arms, legs, and shoulders without premonitory urge. She reported pain and numbness in the left side of her head and in the tongue. Fine motor tics were observed around the eyes. During sleep, the patient’s limbs were observed to be jerking, and she screamed and reported nightmares. Vocal tics and breathing tics were also reported. She described the headaches as a sense of electric shocks or cold water being poured over her head. She also described muscle weakness and feeling exhausted. The feeling of numbness of the left side of her body continued and has never fully disappeared. She had difficulties concentrating and writing and could not stop herself from repeating sentences over and over. She was diagnosed with tic disorder and was sent home with a prescription of analgesics (acetaminophen). Although the symptoms persisted, she was considered healthy apart from the tics. A child psychiatrist was engaged for further therapy.

- ***Age 18:*** *Possible pediatric autoimmune neuropsychiatric disorder (PANDAS)*

In a follow-up in May, she described episodes of weakness in the left side of her body, difficulty walking, sleep paralysis, a need to constantly sing or dance, and uncontrollable attacks of rage. Because of the PANDAS-like symptoms, anti-streptolysin O was analyzed, which gave a positive result of 1.280 international units/ml (IU/ml). Treatment with penicillin V (1.6 g three times daily) was prescribed for 14 days, and after an initial deterioration during the first week, she improved remarkably. She was able to concentrate again, could participate in school, and the tics had subsided by June. She remained well for approximately 3 months. Thereafter, the symptoms gradually reemerged, without signs of tonsillitis. In addition, she developed panic attacks and agoraphobia and was prescribed the selective serotonin reuptake inhibitor (SSRI) citalopram (60 mg/d) in October.

- ***Age 19:*** *Conversion disorder, psychosis, obsessive-compulsive disorder, or an immunopathy*

The patient developed severe bouts of pain lasting for up to 1 h (*see* Additional file 1: Video 1), left-side weakness, difficulties walking, foggy vision, sleep disturbances, depression, and anxiety. Occasionally, she was unable to use her left arm and hand; these episodes lasted several days. Because of the chronicity of pain, penicillin V was again prescribed, but this time without improvement. Accordingly, the patient was referred to the adult neurology department in May for evaluation. After 2 weeks of inpatient care, which included clinical examination and a second brain MRI examination with and without contrast, as well as a sleep EEG, which were all interpreted to be within normal range, she was considered to have a “conversion disorder.”


Additional file 1: **Video 1.** Pain attack: Pain attack in the left thigh, combined with a severe headache attack. Also shown are automatic piano-playing movements in the fingers beyond the patient’s control. The patient is rocking to distract herself from the pain sensations. (MP4 6789 kb)


However, during the autumn, her condition deteriorated, and she developed psychotic symptoms, including visual and auditory hallucinations. She could actually see and describe them, and the voices spoke to her and were frightening. She experienced attacks of amnesia and confusion, and once she could not find her way home from the underground station and had to be picked up by her brother. Occasionally, she lost the ability to read or found text incomprehensible, and she would read sentences backward. Her own writing turned illegible. According to her mother, she could speak endlessly without an understanding of her own words. She had recurrent attacks when she would throw things around or pull out all her things from drawers on the floor, and she also had outbursts of rage. Each of these attacks would continue for several minutes. They started with a sense of pain and resulted in shivering, shattering teeth, clenching teeth, screaming and crying, or baby talk. At times, she was completely unresponsive and mute. Intolerance to noise and blurry vision in her left eye were also reported. Haloperidol, an antipsychotic drug, only made her symptoms worse. She used crutches to support her walking on a weekly basis and could no longer attend school. She slept in her mother’s bed and refused to turn the light off at night because of fright. She regularly stayed in bed during the daytime and felt exhausted. She also had obsessive-compulsive symptoms, predominantly checking, hoarding, and washing. The symptoms followed a relapsing-remitting course for several years, with exacerbation when she had caught a cold. In between, she succeeded in finishing her studies at the gymnasium level, and she attended courses at the university level (including, for example, psychology), but she did not pass any examinations.

- ***Age 21:*** *Body jerks, involuntary movements, loss of strength on the left side*

In August, body jerks, involuntary movements in the face and abdomen, and loss of strength on the left side of her body prompted the patient to seek help at the neurology emergency unit. Due to lack of objective somatic signs, she was still considered as having a psychiatric disorder and was recommended to contact the psychiatric department.

- ***Age 22:*** *Pediatric autoimmune neuropsychiatric disorder reassessed*

The patient’s condition significantly deteriorated following dental root canal work, and in October, she was examined at the department of psychiatry. She made a good impression as a polite, cheerful, and well-groomed young woman who seemed remarkably untroubled, considering the severity of the communicated symptoms. A total of 62 different symptoms were reported, some of which are included in the following list: psychiatric symptoms (hallucinations, paranoid references, obsessive-compulsive symptoms, hoarding, anxiety, sleep disturbance, depression), vegetative symptoms (urinary frequency, nausea, loose stools), neurocognitive symptoms (attention deficit, alexia), perceptual symptoms (numbness, things appeared to be placed farther away than they actually were, hands felt alien, sensation that someone was close by), motor symptoms (dysgraphia, rocking, hyperactivity, grimacing, balance problems, difficulties with walking, falling, cannot hold knife and fork), dull pain (left side of the face, headache, back, feet). Over the years, she had also gained in weight from a BMI of 17.7 kg/m^2^ at age 17 to BMI 25.4 kg/m^2^.

The patient was assessed with structured interviews for PANDAS using the Pediatric Acute Neuropsychiatric Symptom Scale [4], psychiatric rating scales for obsessive-compulsive disorder [5], tics [6], social anxiety disorder, schizotypal personality [7], and depression and neurological soft signs [8]. For all measures, she received scores that suggested pathology (Brief Obsessive-Compulsive Scale, 9 points (range, 0–24); the Yale Global Tic Severity Scale, 12 points (range, 0–30); the Liebowitz Social Anxiety Scale self-report, 90 points (range, 0–144); Schizotypal Personality Questionnaire, 41 points (range 0–74); and the Neurological Evaluation Scale, 46 points (range, 0–84).

Immunological markers analyzed from serum [9] showed elevated Ca^2+^/calmodulin-dependent protein kinase II (CaM kinase II) activity (132%) and D1 and D2 receptor antibodies (titers 8000 and 32,000) (Table 1). Moreover, neuropsychological assessment revealed impaired visuospatial functioning and signs of cognitive deterioration with a full-scale IQ of 87. Oral corticosteroids (prednisone; topical Deltasone, Hikma Pharmaceuticals, London, UK) were tested for 3 days but resulted in further deterioration.

**Table 1:** Biological markers in serum and cerebrospinal fluid

Year	Age (years)	Source	AQP4	NFL	CXCL13	CaMK II	D1	D2	MOG	Alb quota
2012	22	Serum	–	–	–	**132**	**8000**	**32,000**	–	–
2012	22	CSF	–	–	–	**209**	250	250	–	3.7
2014	24	Serum	**1:100**	–	–	109, 99	Negative	Negative	Negative	–
2014	24	CSF	Negative	300	**44**	–		–	Negative	3.6
2015	25	Serum	**1:10**	–	–	–	–	–	**1:32**	–
2015	25	CSF		**400**	**167**	–	–	–	Negative	4.4
2018	28	Serum	**1:10**	–	–	–	–	–	–	–
2018	28	CSF	Negative	370	3	–	–	–	–	3.1

*Abbreviations: Alb quota* Ratio between albumin concentration in CSF (mg/L) and serum (g/L) (reference < 6), *AQP4* Aquaporin-4 antibody, NMO Neuromyelitis optica (IgG) (reference negative), *NFL* Neurofilament light protein (reference < 380), *CSF* Cerebrospinal fluid, *CXCL13* Chemokine (C-X-C motif) ligand 13, also known as B-cell-attracting chemokine 1 (BCA-1) (reference < 7.8), *CaMK II* Ca^2+^/calmodulin-dependent protein kinase II (positive > 130%), *D1* Dopamine receptor antibody positive > 2000, *D2* Dopamine 2 receptor antibody positive > 8000, *MOG* Myelin oligodendrocyte glycoprotein (IgG) (reference negative)

Positive values are presented in bold

Laboratory tests showed low hemoglobin (112), slightly elevated serum neuron-specific enolase (12), and low levels of vitamin D (17 nmol/L), but the other laboratory findings were normal, including S-100B and the anti-streptolysin O test. Blood and urine tests for acute porphyrias were within normal range. The same laboratory panel analyzed in the CSF showed elevated CaM kinase II activity (209%), although the antibody titers against D1 and D2 and Lyso-GM1 receptors; the albumin quota (3.7) (Table 1); and the CSF cytokines interleukin (IL)-1β, IL-6, IL-8, and tumor necrosis factor were all within the normal range.

Flow cytometric analysis of CSF showed higher numbers of AQP4, synaptotagmin-1 (SYT1), antihistone H3 (H3Cit), chitinase domain-containing 1 (CHID1), and phosphatidylserine (PS) microparticles (MPs) than in six healthy control individuals (Table 2).

**Table 2:** Microparticle numbers of antibodies in last portion of cerebrospinal fluid in our patient and six healthy control individuals (four females)

Individuals	AQP4	SYT1	H3Cit	CHID1	PS
Our patient	446	299	26	1275	768
Healthy control individuals*	64 ± 50.3	59 ± 12.4	12 ± 2	447 ± 137.3	355 ± 120

*Mean ± SD

○ **AQP4** Aquaporin-4, aquaporin 4-fluorescein isothiocyanate (AQP4-FITC, epitope amino acids 273–291). These data indicate that the regulation of vesicle mobility is an important mechanism to alter the delivery/retraction ratio of AQP4 vesicles to/from the astroglial plasma membrane

○ **SYT1** Synaptotagmin-1 encoded by a gene located on chromosome 12q21.2 is an integral membrane protein of synaptic vesicles serving as sensors in the process of vesicular trafficking and exocytosis

○ **H3Cit** Antihistone H3 (citrulline R2 + R8 + R17) antibody - chromatin immunoprecipitation grade (ab5103) measures exposed histone H3 (H3cit), derived most likely from neutrophil extracellular traps (NETs), which are fibrous networks that protrude from the membrane of activated neutrophils

○ **CHID1** Chitinase domain-containing 1, CHID1-FITC amino acids 286–315, article number LS-C208436-200, is a regulator of the inflammatory response by macrophages

○ **PS** Lactadherin (lactadherin-FITC; Haematologic Technologies, Essex Junction, VT, USA) measures the number of phosphatidylserine-exposing particles. An “eat me” signal and a general indicator of apoptosis and immune defense, phosphatidylserine (PS) is used to estimate the overall immune reaction

*Methodology of microparticle assays*: Microparticles (MPs) were measured on a Gallios flow cytometer (Beckman Coulter Life Sciences, Indianapolis, IN, USA). The MP gate was determined using Megamix-plus FSC beads (BioCytex, Marseille, France), which consist of a mix of beads with diameters of 0.1, 0.3, 0.5, and 0.9 μm. The threshold was based on forward scatter, and MPs were defined as particles less than 1.0 μm in size and positive for the specific antibody of aquaporin 4-FITC (AQP4), SYT1-FITC (LSBio Inc., Seattle, WA, USA), antihistone H3, citrulline R2 + R8 + R17 (H3Cit), CHID1-FITC amino acids 286–315, article number LS-C208436-200, and lactadherin-FITC (PS). Results are presented as number of MP events in the MP gate during 45 s of measurement. For details of methodology, see Mobarrez *et al.* [16]

**Fig. 1:**
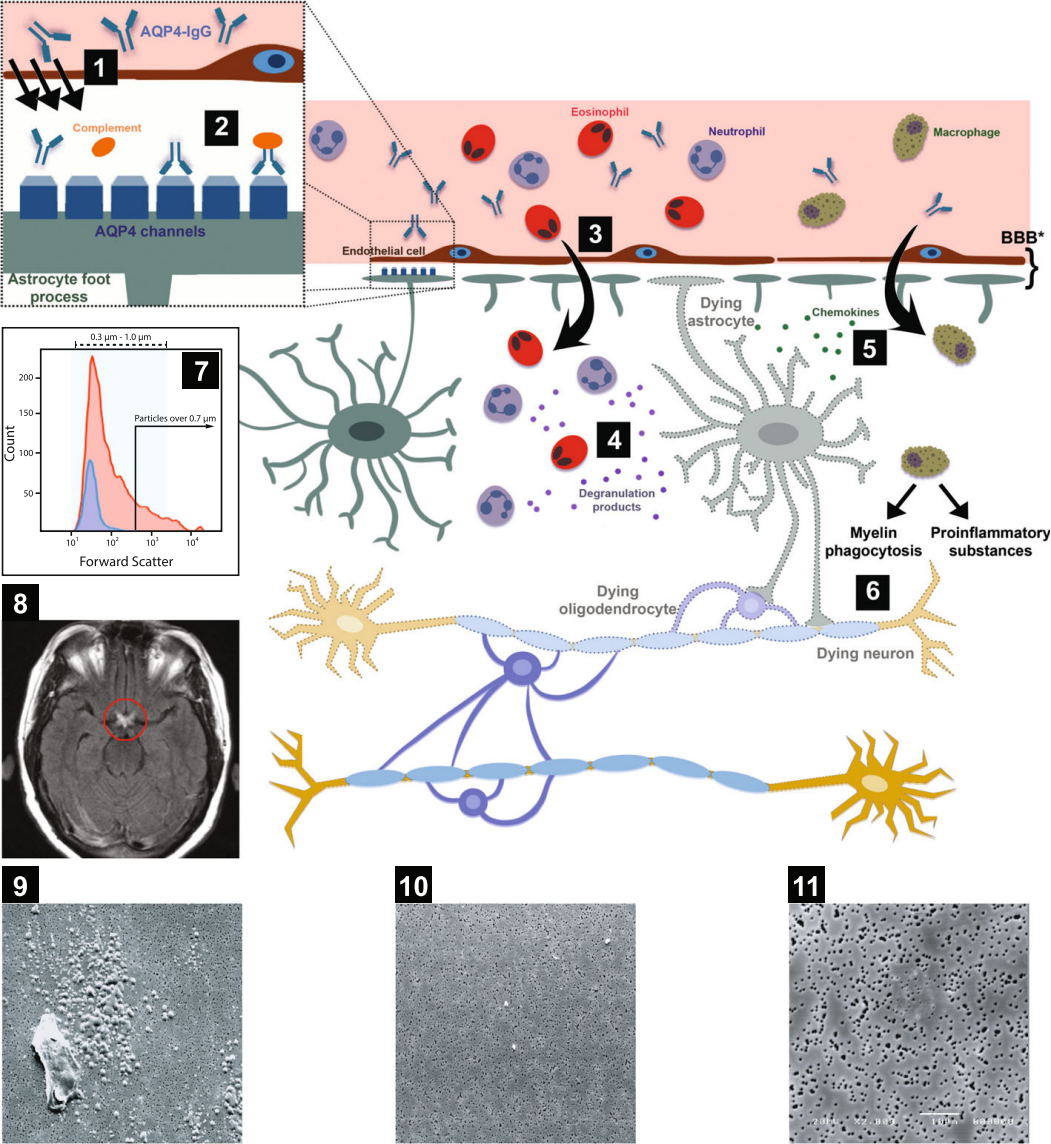
Pathogenesis of neuromyelitis optica spectrum disorder (NMOSD) (**1**–**6**) and clinical findings for the patient (**7**–**11**). **1** Aquaporin-4 immunoglobulin G (AQP4-IgG) accesses the central nervous system at areas of increased blood-brain barrier (BBB) permeability or injury or across endothelial cells by transcytosis. The antibody binds selectively to AQP4 antigen on astrocyte foot processes. The BBB is formed by various components, some of which are illustrated: endothelial cells and astrocyte foot processes. **2** The antigen-antibody binding leads to complement activation and downregulation of the AQP4 water channel. **3** Activated complement increases BBB permeability and leads to leukocyte infiltration, particularly neutrophils and eosinophils. **4** Leukocyte degranulation results in astrocyte death. **5** Chemokines are released from leukocytes and dying astrocytes and attract macrophages. **6** Macrophages produce proinflammatory substances and phagocytose the myelin, resulting in the death of oligodendrocytes (myelin-producing cells) and neurons. **7** Microparticle (MP) numbers of antibodies to AQP4 in the cerebrospinal fluid of the patient at 22 years of age (*red*) compared with a healthy control individual (*blue*). Forward scatter on the *x*-axis indicates the size of the particles, and the total number of MPs is depicted on the *y*-axis. **8** Magnetic resonance imaging of the brain of our patient, showing inflammation (*circled in red*) of both optic nerves as they come together to form the optic chiasm structure. **9** Scanning electron microscopy (SEM) photograph of the very first four drops of cerebrospinal fluid, dropped directly on the polycarbonate filter, displaying a macrophage surrounded by numerous glycogen-like particles. **10** SEM photograph of the last four drops of cerebrospinal fluid shows a few spherical particles with a diameter of about 1 μm. **11** Photograph of cerebrospinal fluid from a healthy control individual does not reveal any specific particles. The diameter of the pores in the filter is 0.6 μm. Cartoons **1**–**6** are adapted with permission from Dutra BG, da Rocha AJ, Nunes RH, *et al.* Neuromyelitis optica spectrum disorders: spectrum of MR imaging findings and their differential diagnosis. Radiographics. 2018;38(1):169–93

Scanning electron microscopy (SEM) of CSF showed spherical particles corresponding to the size of 1 μm in diameter at age 22 years, and the first drops of CSF 6 years later displayed a macrophage with possible glycogen metabolites not present in the last few drops of CSF (Fig. 1).

- ***Age 23:*** *Mixed dissociative syndrome and tonsillitis*

Because of the clinical symptoms and observations, the patient was referred to the neurology department for further examination and treatment. She was reexamined in the spring with a follow-up 4 months later. She was formally diagnosed with a mixed dissociative syndrome, although an organic disorder could not be ruled out. Except for an SSRI for panic attacks and obsessive-compulsive disorder, no treatment was recommended. Tonsillectomy was suggested as a preventive measure to avoid recurrent streptococcal infection, and an operation was performed in the autumn. She developed severe movement symptoms after surgery, before being fully awakened. Hospital personnel were alarmed and called the patient’s mother to watch over her. The movements were filmed (Additional file 2: Video 2). These stereotypies abated, and within a couple of weeks the patient’s physical and psychiatric symptoms improved considerably. She was able to work as a nurse assistant during the following spring.

- ***Age 24*** **:** *AQP4 antibodies indicate NMOSD*

From mid-September, the patient’s physical condition deteriorated, and she became depressed and desperate. At an examination in October, she could barely move her left arm and hand. In a Romberg stance test, she presented choreatic movements in the abdomen and exhibited the “milkmaid’s grip,” which may be seen in Sydenham chorea. She reported episodes of fever, nausea, and difficulties in controlling her left leg. Her left arm felt numb, heavy, and alien (as if it were not her own arm). She fell repeatedly and occasionally needed crutches. She reported poor sleep and nightmares and experienced severe headache. Her boyfriend and mother noted one-sided facial droop during the headache attacks and worried that she might have developed a stroke. By now, the patient’s psychotic symptoms had subsided, and her urinary problem was under control. She was unable to work or study, and she performed worse in the cognitive examination, particularly the visuospatial tests (Rey Complex Figure Test) (Additional file 3: Table S1).


Additional file 2: **Video 2.** Postsurgery: At age 23, the patient had a tonsillectomy to avoid recurrent streptococcal infection. After surgery and before fully waking up, she developed myoclonus jerks in the chest and piano-playing movements in the toes (not shown). (MP4 19799 kb)


In the continued search for biological markers explaining her symptoms, we found that both serum and CSF antibodies were increased for AQP4 with titer 1:100 as a sign of NMOSD (Table 1). On the same occasion, her serum antibodies were negative for GAD65, voltage-gated potassium channel, amphiphysin, CV2, Hu, Ma, Ri, Yo, AMPA (α-amino-3-hydroxy-5-methyl-4-isoxazolepropionic acid), CASPR2, γ-aminobutyric acid B receptor, LGI-1 (leucine-rich glioma inactivated 1), *N*-methyl-d-aspartate receptor, and glycine receptor. She was referred to the university neurology clinic for help with a list of 62 reported clinical symptoms to confirm or exclude the NMOSD diagnosis.

In November, a third CSF examination was performed at the neurology department. The patient’s C-X-C motif chemokine ligand 13 (CXCL13) titers were elevated, as were antibodies toward β-tubulin (Table 1). The CSF/serum ratio was within normal range, and there was no CSF increase of antibodies for myelin oligodendrocyte glycoprotein, glial fibrillary acidic protein, neurofilament light protein (NFL), or AQP4, or against the dopamine receptors D1, D2, and lyso-GM1. CaM kinase II activity was normal at this examination. The patient’s CRP level was still below 1. A planned MRI examination of the spinal cord was postponed because the patient became pregnant in the meantime.

- ***Age 25:*** *Neuritis optica treated with methylprednisolone and B-lymphocyte antigen inhibitor*

A year later, in December, and 2½ months after an uncomplicated delivery of a healthy baby, she experienced severe pain in her left eye, vision loss, and loss of color vision. An ophthalmologic examination showed edema of the papilla and vision of 0.01 in the left eye. She was immediately treated with methylprednisolone (SOLU-MEDROL; Pfizer, New York, NY, USA) 1000 mg three times daily for 3 days. A new MRI scan of her brain and spinal cord revealed swollen optic nerves bilaterally without any other obvious CNS pathology seen.

The CSF sample contained 13 monocytic cells/μl and elevated NFL protein level, but the results of other tests done with the CSF were within the normal range. Treatment with rituximab (Mabthera, Roche) to target the B-lymphocyte antigen CD20-activated glycosylated phosphoprotein on the surface of the cells was initiated in December. In addition to rituximab, daily vitamin D was prescribed. In addition, the patient received cognitive behavioral therapy for her obsessive-compulsive symptoms, with some benefit. However, her cognitive decline persisted, and her visuospatial impairment was further aggravated compared with the previous examination at age 23.

- ***Age 26:*** *In the spring, the patient’s eyesight was fully restored.*

At follow-up 4 months later, the patient’s eyesight was fully restored. However, she reported numbness and displayed a left patellar hyperreflexia with no other neurological signs or symptoms.

- ***Age 27:*** *Analysis of CSF microparticles for AQP4, antihistone 3, and lactadherin*

By February of the next year, she had received three doses of rituximab. Almost all of her somatic symptoms had subsided. However, her obsessive-compulsive symptoms reoccurred about 1 month prior to the next rituximab treatment and abated 2–3 weeks after each rituximab infusion. The infusions were therefore administered with 4-month intervals. She had no somatic exacerbations during the year, but some cognitive abnormalities remained (such as a sense that when she read a text out loud it sounded “wrong,” and she sensed she had lost her ability to speak English properly). Moreover, a progressive deterioration in performance on a visuospatial test (block design) was noted. However, she nevertheless felt better than she had the previous year. She lived in a stable relationship with her husband and was able to independently care for her child. She was also able to reinitiate her university studies. During this year, she became pregnant again and gave birth to a second healthy child in December. Her diagnosis at this time was NMOSD.

- ***Age 28***

The patient continues treatments with rituximab every fourth month. Almost all obsessive-compulsive symptoms subsided, and at this time point, she did not fulfill diagnostic criteria for any psychiatric disorder. However, a single somatic symptom remained: She experiences brief periods of numbness in her left limbs lasting approximately 30 min about twice weekly, but without affecting her overall function in any way. Her headache had diminished. Her BMI had increased to 32 kg/m^2^. She aims to start a diet and continue with her studies. She reports that she feels well and happy and manages the family without assistance. Both children were reported to have normal development.

## Discussion

We report a case of a young woman with a multisymptom presentation of the rare autoimmune disorder NMOSD. At age 24 years, her AQP4 autoantibody titers in serum were clearly increased above reference level. The diagnosis of NMOSD was later confirmed by indication of swollen optic nerves on MRI, and she exhibited a positive treatment response to a monoclonal B-cell inhibitor.

Our patient’s symptoms went etiologically undiagnosed for several years, even though plenty of neuropsychiatric examinations were conducted. The main explanation for the diagnostic delay was that no clear clinical and biological indicators were found. Physical signs, however, such as anorexia resulting in low BMI, as well as the pain pattern described in this report, are signs of an emerging NMOSD but may also occur in PANDAS.

### Conversion disorder

When no established biological indicator can be identified, and if the symptoms are inconsistent with recognized somatic disorders, a neurological disorder is often rejected. Instead, nonsomatic diagnoses such as conversion disorder and dissociative syndrome tend to be used for diagnostic purposes. Conversion disorder, as stated in the *Diagnostic and Statistical Manual of Mental Disorders, Fifth Edition* [10], includes one or more deficits affecting either motor or sensory function that are incompatible with clinically recognized neurological or medical conditions; cannot be explained by any other medical or mental disorder; and cause clinically significant distress or impairment in occupational, social, or other important areas of functioning. Our patient was in fact aware of her happy and untroubled appearance. She said that she felt sad but could nevertheless not resist smiling and laugh when she meant to cry. She did not present maladaptive personality traits, but rather the opposite, which is inconsistent with a conversion disorder. She was concerned about her physical condition and felt depressed, which speaks against a conversion disorder diagnosis. Also, she reported no history of childhood neglect or abuse, which is thought to be overrepresented in conversion disorder.

### *La belle indifférence* and eye movement disorder

Possibly, the “*la belle indifférence*” appearance in our patient could be a sign of an ongoing immune neuropsychiatric disorder rather than a sign of a “hysterical” reaction associated with conversion disorder. *La belle* indifférence may also be an expression of a lack of mimic due to eye movement disorder in the context of neurological illness [11].

### Testing for autoantibodies of AQP4

With the documented myoclonus seen in Additional file 1: Video 1 and Additional file 2: Video 2, we tested for autoantibodies against AQP4, which is located in the astrocyte membrane.

### Microparticles in cerebrospinal fluid

In retrospect, we tested for more MPs in the CSF, which was sampled when the patient was 22 years old. MPs (or microvesicles) carry identity proteins and bioactive molecules from parental cells. Their detection and identification in the CSF may be considered as a direct indicator of activation or damage from specific cells or tissues [12].

Our data suggest a special role for CSF AQP4 MPs as autoantigen-expressing elements capable of perpetuating formation of inflammatory AQP4 autoantibodies. Because of the symptoms of psychosis with hypothetically overactive synapses, we examined synaptotagmin-1 MPs, which were also increased, indicating a dysfunction of synaptic vesicular trafficking. The increased numbers of antihistone H3 (H3Cit) MPs in CSF in the early phase of the disease process may indicate an ongoing vascular defect in the CNS [13].

CHID1 indicates an increased inflammatory response by macrophages. Phosphatidylserine-containing MPs, as well as the protein chemokine CXCL13, were both expressed in the CNS as a sign of an inflammatory response. CXCL13 seems to be the major determinant for B-cell recruitment to the CNS compartment in different neuroinflammatory diseases, rather than being specific for a particular disease entity [14].

Other immunoreactive complement regulators have been reported to protect peripheral organs, but not the CNS, from AQP4-IgG and complement-mediated damage [15]. This finding may explain why NMODS primarily damages the CNS but spares peripheral organs.

One take-home message from this report is that AQP4 autoantibodies found in NMOSD might be triggered by an increase in AQP4 MPs, which may precede the neuropsychiatric symptoms related to optic neuritis.

## Conclusions

There is as yet no consensus on the precise etiology of NMOSD. Testing for more antibodies of AQP4 epitopes for MP analysis in CSF may be useful in differentiating clinical subtypes of patients with NMOSD, as well as for diagnosing other neuropsychiatric diseases where the pathogenesis of the disease remains unknown. Further research in this area is warranted. There are a number of diseases in medicine that, despite being rare, have led to a significant increase in knowledge of human physiology and behavior. This has not been due solely to the unique clinical characteristics that the disease may exhibit, but predominantly from the insight that if the nature of the disease could be explained, it would enable a much broader understanding of the fundamental character of a number of other related diseases. NMOSD disorders may be examples of such a rare disease.

## Additional files

Additional file 3: **Table S1.** Neuropsychological testing of the patient at ages 23, 25, and 27 years. (DOC 38 kb)

## Abbreviations

AQP4: Aquaporin-4
BMI: Body mass index
CNS: Central nervous system
CRP: C-reactive protein
EEG: Electroencephalography
IgG: Immunoglobulin G
IL: Interleukin
MP: Microparticle
MRI: Magnetic resonance imaging
NFL: Neurofilament light protein
NMO: Neuromyelitis optica
NMOSD: Neuromyelitis optica spectrum disorder
PANDAS: Pediatric autoimmune neuropsychiatric disorders associated with streptococcal infections
SSRI: Selective serotonin reuptake inhibitor
TNF: Tumor necrosis factor
