# Supplementary material for: Neuromyelitis optica spectrum disorder with increased aquaporin-4 microparticles prior to autoantibodies in cerebrospinal fluid: a case report
Source: J Med Case Rep. 2019 Jan 30;13:27. doi: 10.1186/s13256-018-1929-z (PMC6352324; doi:10.1186/s13256-018-1929-z)
Supplement: Supplementary file 3 — Table S1. Neuropsychological testing of the patient at ages 23, 25, and 27 years. (DOC 38 kb) [file 13256_2018_1929_MOESM3_ESM.doc]

**Table S1 Neuropsychological testing of patient M at age 23, 25 and 27 years.**

M has been tested with parts of WAIS on three occasions. The testing was performed in Swedish. M is fluent in Swedish but it is not her mother tongue. Since language and school participation affect some tests of WAIS, namely Vocabulary and Information, these results should be interpreted with some caution. See table S1 for full results.

Notes from January 2013: M provides a very diverse profile. Full scale IQ was estimated to be 88, which corresponds to be almost 1 standard deviation (SD=15) below the population mean (m=100). There was a very large and unusual difference between the lowest score of 4 in Similarities and the highest score of 12 in Block design. Notably, M displayed an immature pencil grip during this testing.

In May 2017 Ms estimated full scale IQ (87) is very similar to that of 2013. However, there is a considerable decline in Block-design score over the three occasions. This may be due to the NMOS that M developed. She no longer displays an immature pencil grip and is focused during the length of the testing.

.

**Table S1.** WAIS-III results from three test occasions for patient M at age 23, 25 and 27 years

| Test WAIS-III | Age 23 y | | Age 25 | | Age 27 | |
| --- | --- | --- | --- | --- | --- | --- |
|  | Raw score | Scaled Score | Raw score | Scaled Score | Raw score | Scaled Score |
| Information | 14 | 10 |  |  | 12 | 8 |
| Similarities | 9 | 4 |  |  | 18 | 8 |
| Vocabulary |  |  |  |  | 28 | 7 |
| Comprehension |  |  |  |  | 20 | 10 |
| Arithmetic | 11 | 9 |  |  | 9 | 6 |
| Digit Span | 12 | 6 | 9 | 4 | 12 | 7 |
| Block design | 51 | 12 | 34 | 8 | 24 | 6 |
| Picture completion | 17 | 7 |  |  | 17 | 7 |
| Matrix reasoning |  |  |  |  | 12 | 8 |
| Digit Symbol Coding | 69 | 8 | 69 | 8 | 76 | 10 |
| Symbol search | 29 | 8 |  |  | 34 | 10 |
